# Genetic loci of *Staphylococcus aureus* associated with anti-neutrophil cytoplasmic autoantibody (ANCA)-associated vasculitides

**DOI:** 10.1038/s41598-017-12450-z

**Published:** 2017-09-22

**Authors:** Corinna Glasner, Marcus C. de Goffau, Mirjan M. van Timmeren, Mirja L. Schulze, Benita Jansen, Mehri Tavakol, Willem J. B. van Wamel, Coen A. Stegeman, Cees G. M. Kallenberg, Jan P. Arends, John W. Rossen, Peter Heeringa, Jan Maarten van Dijl

**Affiliations:** 1 Department of Medical Microbiology, University of Groningen, University Medical Center Groningen, Hanzeplein 1, P.O. Box 30001, 9700 RB Groningen, The Netherlands; 2 Department of Pathology and Medical Biology, University of Groningen, University Medical Center Groningen, Hanzeplein 1, P.O. Box 30001, 9700 RB Groningen, The Netherlands; 3 Department of Medical Microbiology and Infectious Diseases, Erasmus MC, ‘s Gravendijkwal 230, 3015 CE Rotterdam, The Netherlands; 4 Department of Internal Medicine, Division of Nephrology, University of Groningen, University Medical Center Groningen, Hanzeplein 1, P.O. Box 30001, 9700 RB Groningen, The Netherlands; 5 Department of Rheumatology and Clinical Immunology, University of Groningen, University Medical Center Groningen, Hanzeplein 1, P.O. Box 30001, 9700 RB Groningen, The Netherlands

## Abstract

The proteinase 3 (PR3)-positive anti-neutrophil cytoplasmic autoantibody (ANCA)-associated vasculitis (AAV) granulomatosis with polyangiitis (GPA) has been associated with chronic nasal *S. aureus* carriage, which is a risk factor for disease relapse. The present study was aimed at comparing the genetic make-up of *S. aureus* isolates from PR3-ANCA-positive GPA patients with that of isolates from patients suffering from myeloperoxidase (MPO)-ANCA-positive AAV, and isolates from healthy controls. Based on a DNA microarray-based approach, we show that not only PR3-ANCA-positive GPA patients, but also MPO-ANCA-positive AAV patients mainly carried *S. aureus* types that are prevalent in the general population. Nonetheless, our data suggests that MPO-ANCA-associated *S. aureus* isolates may be distinct from healthy control- and PR3-ANCA-associated isolates. Furthermore, several genetic loci of *S. aureus* are associated with either PR3-ANCA- or MPO-ANCA-positive AAV, indicating a possible role for pore-forming toxins, such as leukocidins, in PR3-ANCA-positive GPA. Contrary to previous studies, no association between AAV and superantigens was detected. Our findings also show that a lowered humoral immune response to *S. aureus* is common for PR3-ANCA- and MPO-ANCA-positive AAV. Altogether, our observations imply that the presence or absence of particular virulence genes of *S. aureus* isolates from AAV patients contributes to disease progression and/or relapse.

## Introduction

The systemic autoimmune diseases granulomatosis with polyangiitis (GPA) and microscopic polyangiitis (MPA) belong to the anti-neutrophil cytoplasmic autoantibody (ANCA)-associated vasculitides (AAVs)^1^. AAVs are characterized by the presence of circulating ANCAs and inflammation of small to medium-sized vessels, typically affecting lungs and kidneys^2^. The main targets of ANCAs are proteinase 3 (PR3) and myeloperoxidase (MPO), two lysosomal enzymes of neutrophils and monocytes. PR3-ANCAs are predominant in GPA and occur mostly in Northern-European patients, whereas MPO-ANCAs are generally associated with MPA and are mostly occurring in patients in Asia and Australia^2^. Notably, AAVs are multifactorial diseases with numerous contributing genetic and environmental factors^3,4^. Among the latter, microbial upper airway infections have been associated with PR3-ANCA-GPA. In particular, 60–70% of PR3-GPA patients are chronic nasal carriers of *S. aureus*, in contrast to 20–30% of healthy individuals^5,6^. Moreover, PR3-GPA patients carrying *S. aureus* have an increased risk of disease relapse while anti-bacterial treatment reduces the risk of relapse^5,7,8^. Accordingly, particular virulence factors of *S. aureus*, such as the staphylococcal superantigen (SAg) toxic shock syndrome toxin-1 (TSST-1), were previously implicated in PR3-ANCA-GPA disease relapse^9,10^.

We have previously demonstrated that PR3-ANCA-GPA patients carry highly diverse *S. aureus* types that mirror the general *S. aureus* population^11^. Yet, this finding does not rule out the possibility that *S. aureus* carried by PR3-ANCA-positive patients possesses a particular genetic make-up that could contribute to disease progression and/or relapse. Studies on *S. aureus* isolates from MPO-ANCA-positive patients have so far been lacking. Therefore, the present study was aimed at investigating the gene repertoire of *S. aureus* nasal isolates from PR3-ANCA-positive patients and to compare it to that of *S. aureus* isolates from MPO-ANCA-positive patients and from healthy controls (HC). Additionally, the humoral immune response of these two AAV patient groups against a comprehensive set of *S. aureus* antigens was compared, because our previous studies had shown that GPA patient sera contained lower anti-staphylococcal IgG levels than sera from HC^11^.

## Materials and Methods

### Patients, bacterial isolates and serum samples

This retrospective study included *S. aureus* isolates and serum samples from AAV patients and HC with a Caucasian background. All patients fulfilled the Chapel Hill Consensus Conference definitions for the diagnosis of AAV and regularly visited the University Medical Center Groningen (UMCG, The Netherlands)^1^. The patients were selected based on availability of stored *S. aureus* isolates and/or serum samples, but formed a representative cohort of all AAV patients from our hospital.

The 61 investigated *S. aureus* isolates from 32 PR3-ANCA patients and the 18 isolates from 10 HC were collected in the period 2006–2012, and have been described in a previous study (Table 1)^11^. The 27 newly investigated *S. aureus* isolates from 27 MPO-ANCA patients were collected in the same period. Characteristics of the *S. aureus* isolates used in this study are summarized in Table 1. Of note, the sample size of *S. aureus* isolates from MPO-ANCA patients is relatively low, primarily because the number of MPO-ANCA patients in the Netherlands is much lower than the number of PR3-ANCA patients. In addition, *S. aureus* isolates from MPO-ANCA patients are not routinely frozen in our hospital, in contrast to isolates from PR3-ANCA patients. Further, when more than one isolate per patient was included in the analyses, this concerned always different clinical isolates from this patient collected at different time points.

**Table 1:** *S. aureus* isolate characteristics grouped by PR3-ANCA, MPO-ANCA and HC^1^.

	PR3-ANCA isolates (n = 61)	MPO-ANCA isolates (n = 27)	HC isolates (n = 18)
No. of patients	32	27	10
No. of males (%)	22 (69)	11 (41)	4 (40)
Age at time of swab, mean ± SD	55 ± 13	60 ± 13	33.6 ± 11.8
Collection period	2006–2012	2010–2013	2007–2010
*spa*-types (%)	t002 (1.6)	t002 (3.7)	t002 (5.6)
		t012 (14.8)	t012 (16.7)
	t021 (1.6)	t021 (3.7)	t021 (11.1)
	t064 (37.7)	t064 (3.7)	
	t091 (21.3)	t091 (22.2)	t091 (11.1)
	t015 (1.6)	t026 (7.4)	t008 (5.6)
	t072 (3.3)	t065 (3.7)	t024 (5.6)
	t089 (3.3)	t126 (3.7)	t122 (11.1)
	t224 (1.6)	t223 (7.4)	t209 (5.6)
	t451 (1.6)	t362 (3.7)	t230 (5.6)
	t539 (6.6)	t442 (3.7)	t359 (5.6)
	t1246 (3.3)	t711 (3.7)	t2109 (11.1)
	t1265 (1.6)	t888 (3.7)	t6076 (5.6)
	t1361 (1.6)	t1168 (3.7)	
	t1508 (3.3)	t2849 (3.7)	
	t2658 (1.6)	t6365 (3.7)	
	t3092 (3.3)	t13788 (3.7)	
	t4584 (1.6)		
	t12249 (3.3)		
CC^*^ strain assignment (%)	CC5 (14.8)	CC5 (11.1)	CC5 (5.5)
	CC7 (21.3)	CC7 (22.2)	CC7 (11.1)
	CC8 (45.9)	CC8 (14.8)	CC8 (11.1)
	CC22 (1.6)	CC22 (7.4)	CC22 (5.5)
	CC30 (4.9)	CC30 (18.5)	CC30 (38.8)
	CC45 (1.6)	CC45 (14.8)	CC45 (5.5)
	CC1 (3.3)	CC12 (3.7)	CC9 (5.5)
	CC25 (3.3)	CC361 (3.7)	CC20 (11.1)
	CC97 (1.6)	CC101 (3.7)	CC97 (5.5)
	None (1.6)		
*agr* type (%)	I (75.4)	I (66.7)	I (50)
	II (14.8)	II (14.8)	II (11.1)
	III (8.2)	III (18.5)	III (38.9)
	None (1.6)		
No. of *mecA*-positive isolates	0	1	0
No. of *tst*-1-positive isolates	2	5	8
No. of PVL-positive isolates	0	0	0

^1^The 61 isolates from 32 PR3-ANCA-positive patients (denoted PR3-ANCA isolates) and 18 isolates from 10 HC (denoted HC isolates) have previously been described^11,14,26^. In addition, 27 nasal *S. aureus* isolates from 27 MPO-ANCA-positive patients (denoted MPO-ANCA isolates) visiting our outpatient clinic were collected. At first, all isolates were characterized by *spa*-typing^27^, revealing that among the MPO-ANCA isolates the *spa*-types t012, t064 and t091 were predominant, similar to the PR3-ANCA and HC collection (Table 1)^10^. ^*^CC; clonal complexes.

IgG responses of PR3-ANCA and MPO-ANCA patients and HC to staphylococcal antigens were determined in sera from 27 PR3-ANCA patients (15 *S. aureus* carriers, 12 non-carriers; one serum/patient), 38 MPO-ANCA patients (27 carriers, 11 non-carriers; 1 serum/patient) and 18 HC (10 carriers with 23 sera, eight non-carriers with 20 sera).

### Ethics statement

All bacterial isolates and human serum samples were obtained from an already-existing collection of the ‘Groningen cohort of ANCA-associated vasculitis’, for which institutional review board (IRB) approval of the experimental protocols was previously obtained from the Medical Ethics Committee of the UMCG. Written informed consent was obtained from all patients, and all experiments were conducted in accordance with the guidelines of the Declaration of Helsinki. All isolates and samples were anonymized.

### DNA microarray

DNA was isolated with the UltraClean Microbial DNA Isolation Kit (MoBio, Carlsbad, USA). The Clondiag *S. aureus* Genotyping Kit 2.0 (Alere Technologies GmbH, Jena, Germany) was used for DNA microarray analyses^12,13^. This microarray contains 336 DNA probes to detect genes for species-specific markers, antibiotic resistance, SCC*mec* elements, adhesion and virulence factors, capsule and *agr* group markers. The affiliations of isolates to specific multilocus sequence type clonal complexes (MLST CC) were determined by synchronizing the hybridisation profiles to a reference database^12^.

### Multiplex S. aureus antibody assay

Bead-based Luminex flow cytometry (xMAPH, Luminex Corporation, Austin, Texas, USA) was performed as previously described^14^.

### Statistical analyses

Statistical analyses were performed with SPSS Statistics 20 (SPSS, Chicago, USA) or GraphPad Prism (Version 6, La Jolla, California). Principal component analysis (PCA) was used to describe the variation between all three *S. aureus* isolate groups (i.e. PR3-ANCA, MPO-ANCA and HC) into a very limited number of new relevant dimensions in order to address the issue of multiple testing and to identify clusters of similar or different gene profiles. Genes that were universally present or absent in all samples from all *S. aureus* isolate groups were excluded from the PCA as they do not describe any variation. Cluster analysis was performed using the updated Hierarchical Clustering Explorer 3.0 (HCE), which is publically available at www.cs.umd.hcil/hce ^15^. Differences in anti-staphylococcal antibody levels between groups were tested for statistical significance using the Kruskal-Wallis test. A two-sided *p* value < 0.05 was considered to be statistically significant.

## Results and Discussion

We employed DNA microarrays to determine the genetic profiles of all 106 *S. aureus* isolates in the PR3-ANCA, MPO-ANCA and HC sampling groups. The results of these array analyses underscored the previously reported high genetic diversity of PR3-ANCA isolates^11^, and revealed a comparably high genetic diversity for MPO-ANCA isolates. Table 1 summarizes the basic molecular characteristics of the three *S. aureus* sampling groups, and Supplementary Information Table 1 presents all microarray data. As shown in Table 1, the six most common *S. aureus* clonal complexes (CCs) are represented in all three groups of isolates (i.e. PR3-ANCA, MPO-ANCA and HC). These six CCs are in fact the most common CCs in Europe^16–18^. CC8 was predominant amongst PR3-ANCA isolates (~46%), while CC30 and CC45 prevailed amongst MPO-ANCA isolates (~19% and ~15%, respectively). CC5 and CC7 were both common amongst MPO-ANCA and PR3-ANCA isolates. HC isolates belonged mainly to CC30 (~39%) and the remaining 11 (~61%) isolates were assigned to nine different CCs. Of note, in those cases where more than one *S. aureus* isolate per PR3-ANCA patient or HC was investigated, the microarray data showed that this concerned isolates with a different genetic makeup, consistent with the results of *spa*-typing (Table 1).

### Identification of potentially disease-associated staphylococcal determinants

**Figure 1:**
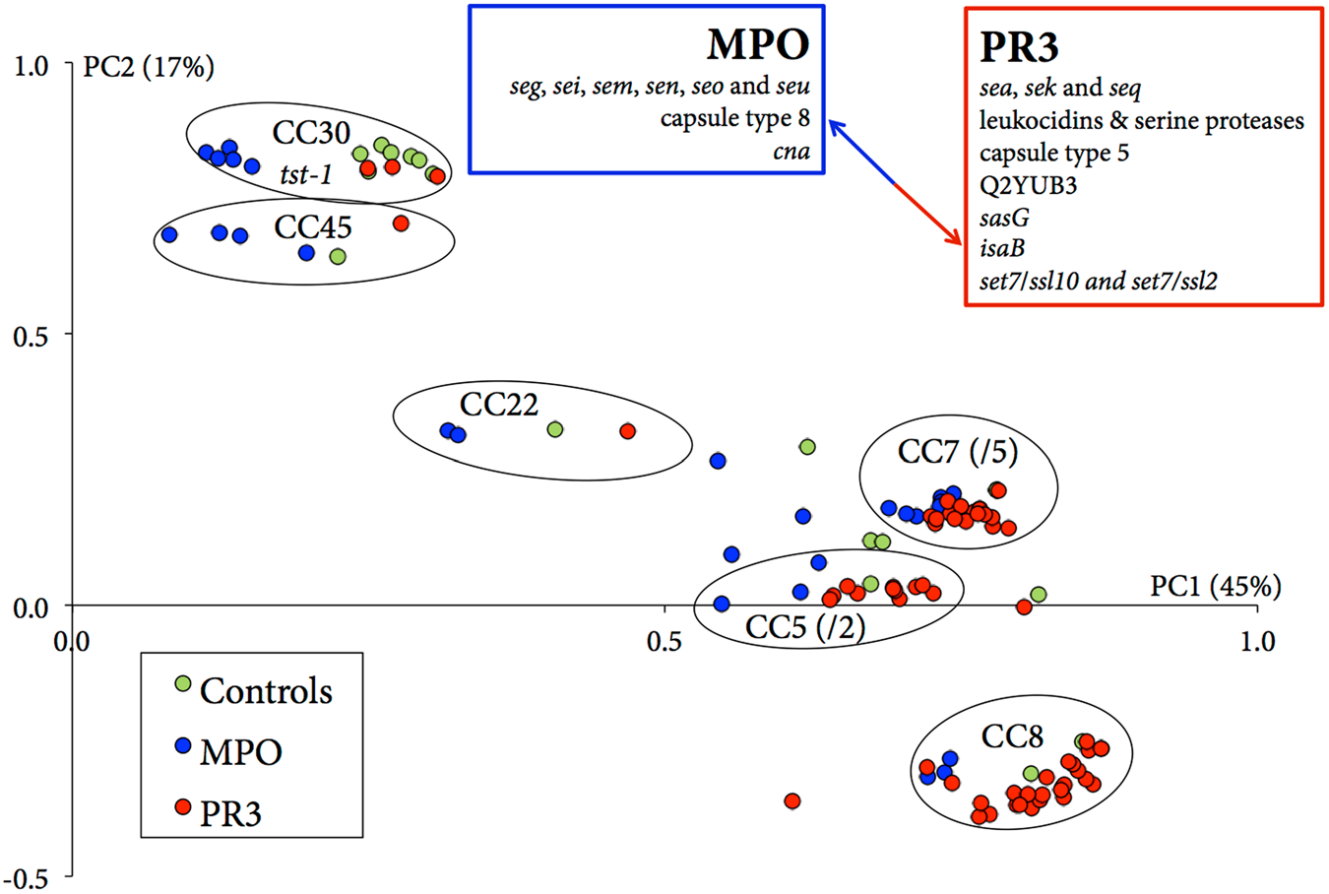
PCA of the ClonDiag microarray data. The x-axis represents PC1 and the y-axis represents PC2, which describes 45% and 17% of all of the variation in the data, respectively. Note that the circle indicating CC7 also includes five isolates that belong to the different low-abundance CC variants. The same is true for 2 isolates in the CC5 circle. The blue and red boxes display single genes or groups of genes that were associated either positively with PC1 and negatively with PC2 (red box), or the other way round (blue box). The associations with PC1 and PC2 are strongly correlated with the different CCs, of which the abundances differ between PR3-ANCA, MPO-ANCA and HC. The localization within each CC circle however also shows a clear intra-CC-specific association of PC1 with either MPO-ANCA (left) or PR3-ANCA and HC (right).

Since the PR3-ANCA, the MPO-ANCA and the HC isolates represented highly diverse *S. aureus* types, a PCA was performed that focused on the genetic variation within the CCs identified in these isolate groups. Importantly, the location of all 106 *S. aureus* isolates on the first two principal components (PCs) already explained 62% of the variation in the data (Fig. 1). As expected, isolates from the same CC were grouped together (Fig. 1, marked with circles). As MPO-ANCA, PR3-ANCA and HC isolates were not evenly distributed amongst CCs, the (indirect) association of certain staphylococcal genes with PR3-ANCA or MPO-ANCA (indicated in Fig. 1) may relate to the association of particular CCs with either of the two disease types.

**Figure 2:**
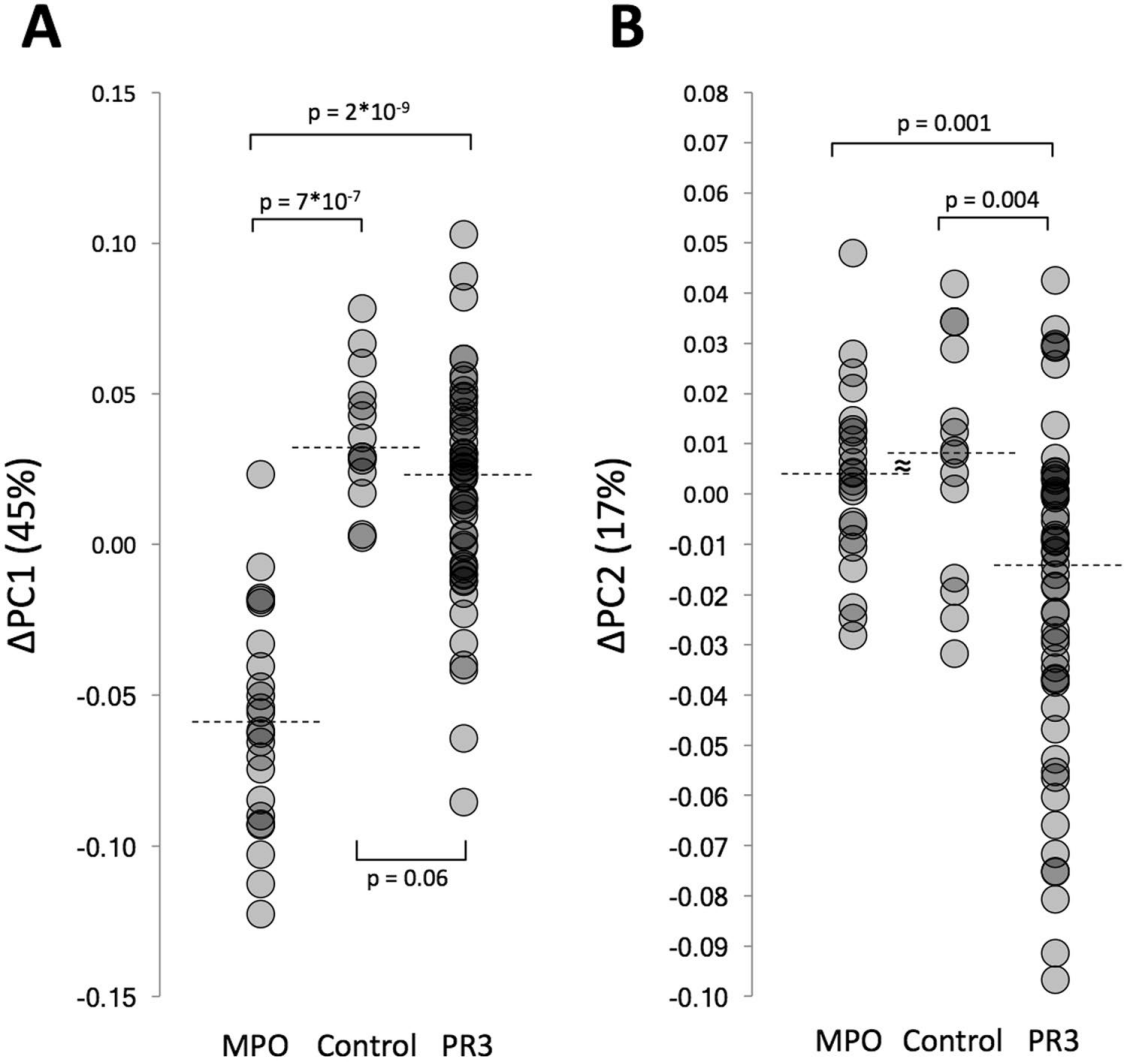
Visualization of the differences in variation (Δ) within each of the six main CCs, as described by ΔPC1 (A) and ΔPC2 (B), of PR3-ANCA, MPO-ANCA and HC isolates. Dashed lines represent median values.

Other associations with either PR3-ANCA or MPO-ANCA, which are CC-independent, can be found by looking at the variation which exists within each clonal complex. For this the average PC1 value of each CC was used to calculate the respective position of each isolate on PC1 in comparison with the rest of the isolates that are from the same clonal complex group (ΔPC1). This is represented in Fig. 1 by isolates being located more to the left or more to the right within a CC circle. PC1 was used for this as it describes nearly half of the variation within the entire dataset (45%). In Fig. 2, it is revealed that MPO-ANCA isolates were negatively correlated with ΔPC1, i.e. these isolates were shifted to the left within each CC cluster (Fig. 1). Conversely, PR3-ANCA but also the HC isolates were positively correlated with ΔPC1, i.e shifted to the right (Fig. 1). This implies that the HC isolates have apparently more in common with PR3-ANCA isolates than with MPO-ANCA isolates.

**Figure 3:**
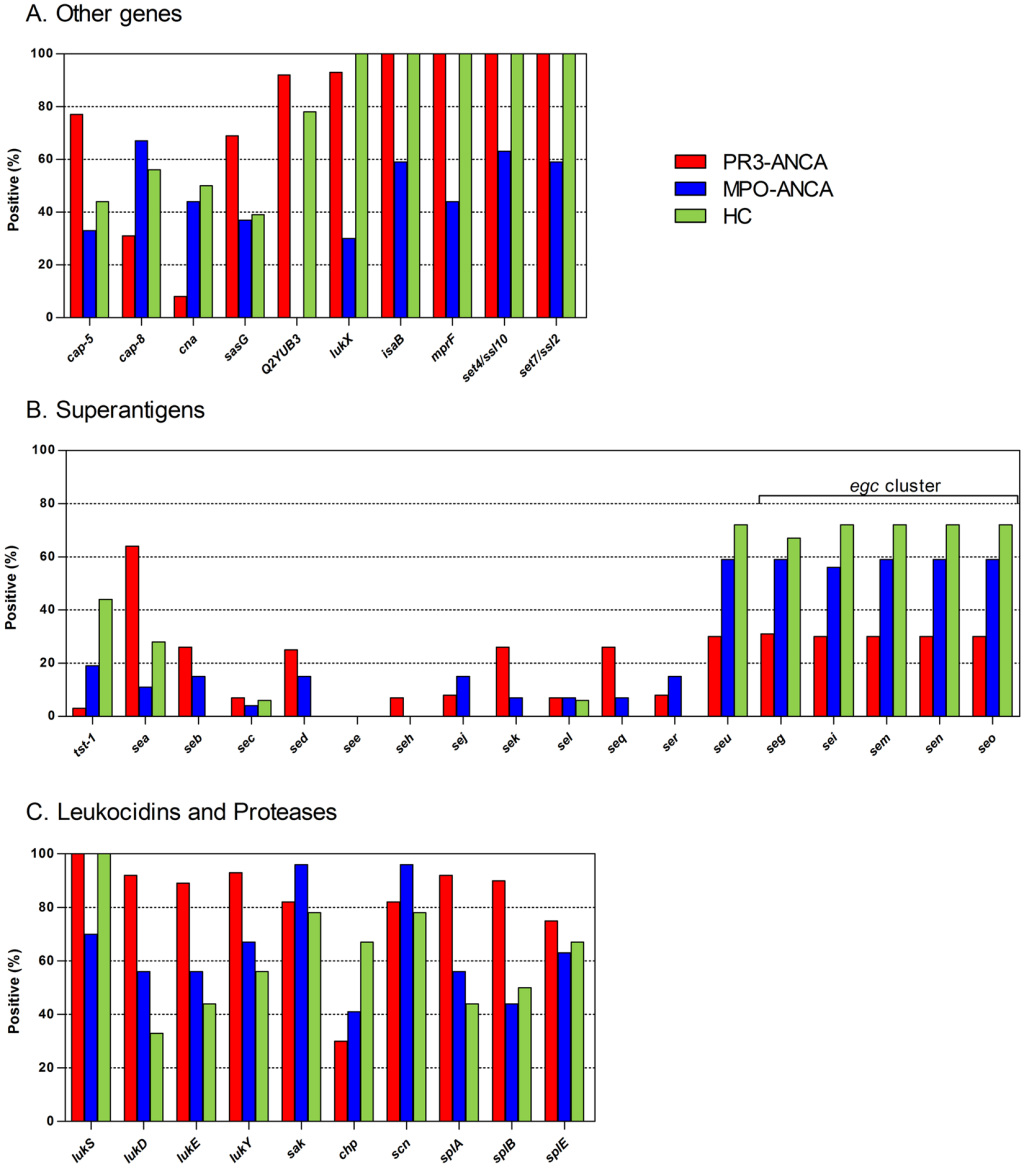
Prevalence of selected genes that were identified before or after correlation analysis of ΔPC1 in PR3-ANCA, MPO-ANCA and HC *S. aureus* isolates. (**A**) The genes *cap*-5, *cap*-8, *can*, *sasG*, Q2YUB3, *lukX*, *isaB*, *mprF*, *set4/ssl10* and *set7/ssl2*, (**B**) superantigen-encoding genes, and (**C**) leukocidin, immune evasion and protease genes.

Correlation analyses with ΔPC1 identified several genes associated with PR3-ANCA and HC isolates, but not with MPO-ANCA isolates (p < 0.001). These genes include Q2YUB3, *lukX*, *isaB*, *mprF*, *set4/ssl*1*0* and *set7/ssl2* (Table 2). Figure 3A displays the prevalence of these six genes *plus* four genes (*cap*-5, *cap*-8, *cna* and *sasG*) that were associated with PR3-ANCA or MPO-ANCA isolates before the correlation analyses of each CC cluster (Fig. 1). Specifically, the *cap*-5, *sasG*, Q2YUB3, *lukX*, *isaB*, *mprF*, *set4/ssl10* and *set7/ssl2* genes were more abundant amongst PR3-ANCA isolates than MPO-ANCA or HC isolates, while the *cap*-8 and *cna* genes were less abundant amongst PR3-ANCA isolates. However, *cap*-5, *cap*-8, *cna* and *sasG* are only associated with PR3- or MPO-ANCA due to the association of the respective CCs with the two disease types.

**Table 2:** Genetic loci of *S. aureus* associated with AAV and functions of the respective proteins.

Gene name	Encoded function
Q2YUB3	multidrug resistance protein
*lukX* ^*^	leukocidin/haemolysin toxin X
*isaB* ^*^	immunodominant antigen B
*mprF* ^*^	probable lysylphophatidylglycerol synthetase (defensin resistance)
*set4/ssl10* ^*^	staphylococcal superantigen-like protein 4
*set7/ssl2* ^*^	staphylococcal superantigen-like protein 7
*cap*-5	capsular polysaccharide protein 5
*cap*-8	capsular polysaccharide protein 8
*cna*	collagen-binding adhesion protein
*sasG*	*S. aureus* surface protein G

^*^Associated with ΔPC1.

**Figure 4:**
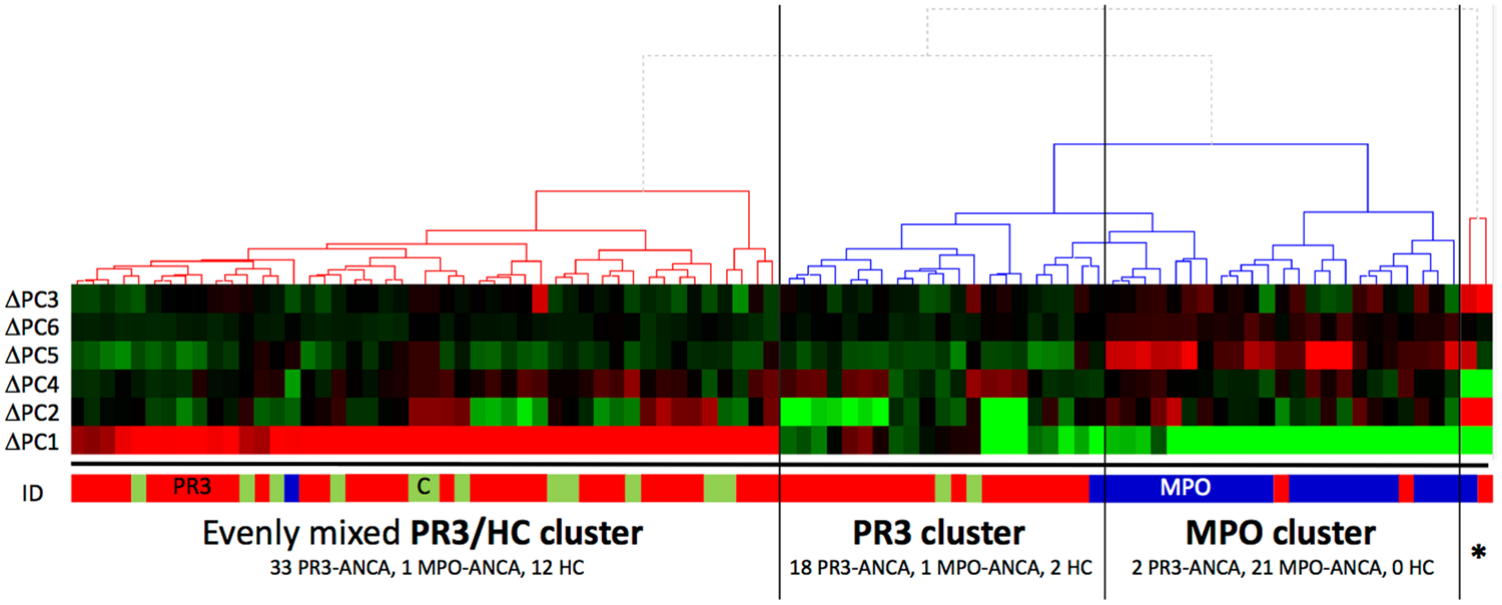
Hierarchical clustering analysis on the variation within each of the 6 main clonal complexes, as described by the first 6 principal components (ΔPC1 – ΔPC6). A complete linkage clustering on the weighted results of ΔPC1 – ΔPC6, which together described 83% of the variation present within the entire data set, separate nearly all of the *S. aureus* isolates up into three unique groups. The small group indicated by *, represents two *S. aureus* isolates from CC8 which differ from the rest of CC8 as they are ST72 MRSA isolates, and hence form an outlier. ΔPC3 (left) does not strongly distinguish PR3-ANCA (red), MPO-ANCA (blue) and HC isolates (green) from one another and hence is represented as the top bar being the least relevant.

Noteworthy, PR3-ANCA isolates can be distinguished to a certain extent from both MPO-ANCA and HC isolates by PCA when subsequently analysing only ΔPC2 (Y-axis, Fig. 2), a vector describing 17% of the variation in the data. A large group of PR3-ANCA isolates score lower on ΔPC2 than all other MPO-ANCA and HC isolates (Fig. 2). A lower score on ΔPC2 is in particular strongly associated with the presence of the *seb*, *sed*, *sek* and *seq* genes, with bovine leucocidin, an arginine/ornithine antiporter, fibronectin-binding protein B and the Q2FXC0 gene (a putative beta-lactamase) (Fig. 3 and Supplementary Information Table 1). When taking most of the variation in the entire dataset together (83%) by doing a hierarchical complete linkage cluster analysis on the weighted results of ΔPC1 to ΔPC6, the MPO-ANCA isolates form a very distinct cluster, whereas most of the PR3-ANCA isolates are indistinguishable from the HC isolates (Fig. 4). A subset of PR3-ANCA isolates forms a distinct cluster in the middle, which sets them slightly apart from the PR3-ANCA/HC cluster on the left because the latter do not score high on ΔPC1 and/or score low on ΔPC2 (Fig. 4). Altogether, the PCA analyses suggest several interesting associations of genes with the PR3-ANCA or MPO-ANCA isolates, especially genes encoding SAgs, leukocidins, hemolysins, enterotoxins and other virulence factors as discussed in the following paragraphs.

### Low prevalence of egc– and non-egc genes for SAgs in the two disease-associated sampling groups

SAgs are secreted toxins that cause non-specific activation and proliferation of T-cells resulting in massive cytokine release. SAgs have therefore been proposed as activators/initiators of various autoimmune diseases^10,19,20^. For PR3-GPA patients it was previously reported that the carriage of SAg-positive or SAg-negative *S. aureus* isolates was equally associated with an increased risk for disease relapse when compared to non-carriage of *S. aureus* ^10^. In the latter study, only the carriage of *tst-1*-positive *S. aureus*, representing 16.5% of the PR3-GPA isolates, was associated with a higher risk for disease relapse. In this context, it is remarkable that only 3% of the presently investigated PR3-ANCA isolates carried the *tst-1* gene (Table 1; Fig. 3B). Conversely, 19% of the MPO-ANCA isolates and even 44% of the HC isolates were *tst-1*-positive (Fig. 3B). In general, ~15–25% of *S. aureus* isolates have been reported *tst*-*1*-positive for different *S. aureus* sampling groups, suggesting that *tst-1* is underrepresented in our PR3-ANCA isolates^16,21^. Furthermore, with respect to the overall SAg gene repertoire of the three *S. aureus* sampling groups, we observed an interesting distribution for *egc* and non-*egc* genes. Both the *egc* cluster, consisting of the five SAg genes *seg*, *sei*, *sem*, *sen* and *seo*, and the non-*egc* gene *seu* were detected in only 30–31% of the PR3-ANCA isolates, but in 56–59% of the MPO-ANCA and even in 67–72% of the HC isolates (Fig. 3B). The remaining non-*egc* SAg genes *sec*, *sed*, *see*, *seh*, *sej*, *sek*, *sel*, *seq* and *ser* were detected in a very limited number of isolates in either of the three sampling groups (Fig. 3B). Only the non-*egc* SAg gene *sea* was abundantly detected in the PR3-ANCA isolates.

Other groups have previously reported remarkable variations in the SAg gene profiles, even within *S. aureus* populations possessing the same CC or *spa*-type^19^. Thus, it is conceivable that the prevalence of SAg genes in the selected *S. aureus* sampling groups reflect this variable distribution of SAg genes. Nonetheless, the presently observed low overall prevalence of *egc*– and non-*egc* SAg genes in PR3-ANCA isolates seems to suggest that TSST-1 and other SAgs may have no critical role in GPA.

### Differential distribution of leukocidins between PR3-ANCA and MPO-ANCA isolates

Previous studies have shown that *S. aureus* has both a variant and an invariant virulence gene repertoire^22^. In accordance with this notion, major *S. aureus* virulence genes for leukocidins and hemolysins (pore-forming toxins), including *lukF*, *hlgA*, *hl*, *hla*, *hld*, *hlIII* and *hlb*, were identified in almost all study isolates (Supplementary Information Table 1). In contrast, all *S. aureus* isolates tested negative for the Panton-Valentin leukocidin (PVL). Intriguingly, differences in the prevalence between the three *S. aureus* sampling groups were observed for several other leukocidin genes, including *lukD*, *lukE*, *lukS*, *lukX* and *lukY*. Firstly, *lukS* and *lukX* were less abundant in the MPO-ANCA sampling group only, and secondly, the *lukD*, *lukE* and *lukY* genes were less abundant in both the MPO-ANCA and HC sampling group (Fig. 3A and C). Overall, *luk* genes were most frequently detected in the PR3-ANCA sampling group. Noteworthy is also the imbalance in the presence of the *lukX* and *lukY* genes in the MPO-ANCA and HC sampling groups, while these genes are present at comparable frequencies in the PR3-ANCA sampling group. This suggests an association between *S. aureus* isolates colonizing PR3-GPA patients and the possible production of the bi-component LukXY leukocidin (designated LukAB^23^ and LukGH^22^). This may be relevant since LukXY has strong cytolytic activity and is able to kill neutrophils, macrophages and dendritic cells^23,24^.

For the immune evasion cluster (IEC), which consists of the *chp*, *scn* and *sak* genes on the so-called *hlb*-converting phage, it was noted that many isolates in the PR3-ANCA and MPO-ANCA sampling groups lacked the *chp* gene for the neutrophil chemotaxis-inhibitory protein, while the *scn* and *sak* genes for the staphylococcal complement inhibitor and staphylokinase, respectively, were abundantly detected in all sampling groups (Fig. 3C). Lastly, the repertoire of protease genes was generally comparable for the three sampling groups, although the prevalence of the two protease genes *splA* and *splB* was higher in PR3-ANCA isolates than in MPO-ANCA and HC isolates (Figs 1 and 3C).

### Low levels of anti-staphylococcal antibodies in MPO-ANCA patients

**Figure 5:**
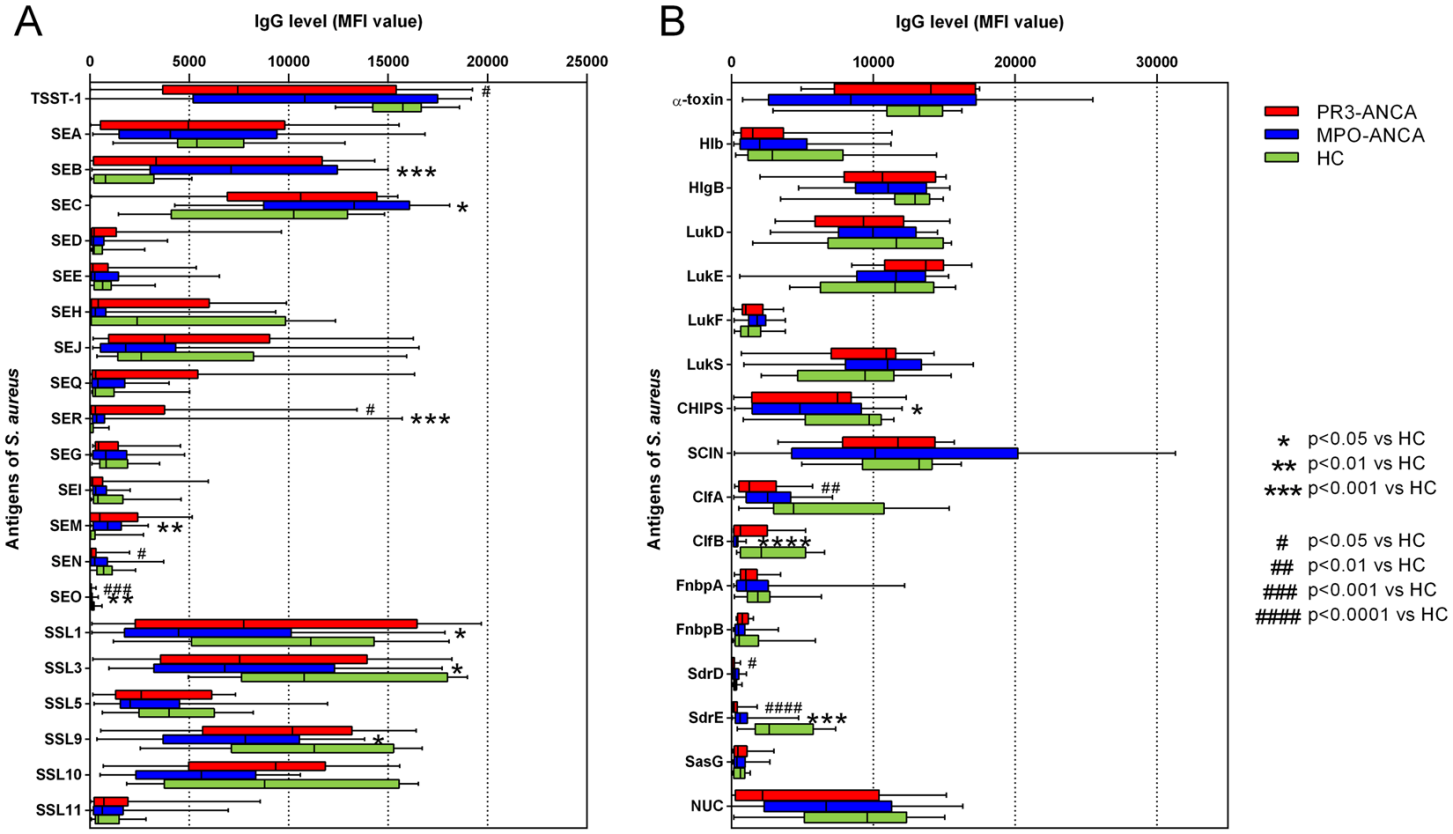
IgG responses of PR3-ANCA and MPO-ANCA patients or HC to staphylococcal antigens. Serum IgG levels against (**A**) secreted superantigens and superantigen-like proteins and (**B**) selected surface and secreted proteins. Depicted are the median with boxes (25% and 75%) and whiskers (minimum to maximum) of all sera per group. Statistical significances were tested using the Kruskal-Wallis test (with post-hoc Dunn’s test). *p < 0.05, **p < 0.01, ***p < 0.001 versus HC and ^#^p < 0.05, ^##^p < 0.01, ^###^p < 0.001, ^####^p < 0.0001 versus HC.

Since PR3-GPA patients seem to have difficulties in mounting a humoral immune response against *S. aureus* ^11^, we also investigated the serum IgG levels of MPO-ANCA patients against 38 *S. aureus* antigens, including 7 surface proteins, 10 secreted proteins and 21 secreted superantigens/superantigen-like proteins. The results were compared to antigen specific serum IgG levels observed for PR3-ANCA patients and HC. As presented in Fig. 5 and Supplementary Figure 1, the overall antibody responses showed a broad variability in all three groups. Importantly, anti-staphylococcal IgG responses were comparable in PR3-ANCA and MPO-ANCA patients, and significant differences could only be detected between these AAV patients and HC. Both MPO-ANCA and PR3-ANCA patients showed lower serum IgG levels against superantigens and superantigen-like proteins (TSST-1, SEN, SEO, SSL1, SSL3, SSL9), and against particular staphylococcal surface proteins (ClfA, ClfB, SdrD and SdrE) than HC, though not always reaching statistical significance. Conversely, IgG levels against the superantigens SEB, SER and SEM were higher in MPO-ANCA patients than in HC as was previously shown for PR3-ANCA patients^11^. It is noteworthy that the relatively low prevalence of the *tst-1*, *sen* and *seo* genes, and the higher prevalence of the *seb* and *ser* genes in the patient isolates is in perfect agreement with the IgG levels against these antigens. Altogether, these findings show that relatively low levels of IgGs against various staphylococcal antigens are a common trait of PR3-ANCA and MPO-ANCA patients.

## Conclusion

The present study provides a first comprehensive portrait of the gene repertoire of *S. aureus* isolates from patients suffering from the PR3-ANCA- and MPO-ANCA-associated forms of the autoimmune disease AAV. Although our retrospective study has some limitations (i.e. relatively low numbers of isolates and patients, or non-matched sampling times), the results show that not only the PR3-ANCA-AAV patients, but also the MPO-ANCA-AAV patients mainly carry *S. aureus* types that are carried in the general population. Nevertheless, our results suggest a different distribution of *S. aureus* CCs amongst these two disease types. More importantly, several genetic loci of *S. aureus* were found to be associated with either PR3-ANCA or MPO-ANCA-AAV. In particular, the *cap-5*, *sasG* and *lukX*-*lukY* genes were found to be positively correlated with the PR3-ANCA isolates. These *S. aureus* genes could thus be potentially involved in PR3-AAV disease. Crucially, our study provides no evidence for a specific SAg profile related to the investigated *S. aureus* sampling groups but, instead, it suggests a possible role for leukocidins in PR3-ANCA-AAV. This is reminiscent of the detrimental effects of these leukocidins on neutrophils from which the PR3 and MPO targets of ANCAs are derived. In this respect, it is noteworthy that the *chp* gene, encoding a protein that impairs neutrophil chemotaxis, was negatively correlated to both PR3- and MPO-ANCA isolates. This ‘guilt by associations’ makes it conceivable that the specific presence or absence of particular genes in *S. aureus* isolates carried by MPO- and PR3-ANCA-positive AAV patients is relevant for disease onset and progression. This could involve a scenario of *S. aureus*-provoked inflammation, where neutrophil chemotaxis is not effectively impaired due to the absence of *chp* leading to enhanced accumulation of neutrophils, in particular in the upper respiratory tract. Here, the neutrophils can be activated by ANCA and release their toxic products leading to acute inflammation characterized by tissue necrosis and microabscess formation. Such an acute neutrophil-rich necrotizing inflammation would evoke a mononuclear leukocyte response promoting granulomatous inflammation, which is typical for GPA^25^. The *S. aureus* cells that provoked the inflammation could potentially survive in this neutrophil-rich environment by producing particular leukocidins.

## Electronic supplementary material

Supplementary Figure 1

Supplementary Table 1
