# Supplementary material for: Genetic loci of Staphylococcus aureus associated with anti-neutrophil cytoplasmic autoantibody (ANCA)-associated vasculitides
Source: Sci Rep. 2017 Sep 22;7:12211. doi: 10.1038/s41598-017-12450-z (PMC5610336; doi:10.1038/s41598-017-12450-z)
Supplement: Supplementary file 1 — Supplementary Figure 1 [file 41598_2017_12450_MOESM1_ESM.doc]

**Supplementary Material**

**Figure 1**

**Genetic loci of *Staphylococcus aureus* associated with anti-neutrophil cytoplasmic autoantibody (ANCA)-associated vasculitides**

Corinna Glasner^1^, Marcus C. de Goffau^1*^, Mirjan M. van Timmeren^2*^, Mirja L. Schulze^1^, Benita Jansen^1^, Mehri Tavakol^3^, Willem J. B. van Wamel^3^, Coen A. Stegeman^4^, Cees G. M. Kallenberg^5^, Jan P. Arends^1^, John W. Rossen^1^, Peter Heeringa^2#^ and Jan Maarten van Dijl^1#†^

^*^These authors contributed equally.

^#^These authors jointly supervised the study.

^1^Department of Medical Microbiology, University of Groningen, University Medical Center Groningen, Hanzeplein 1, P.O. Box 30001, 9700 RB Groningen, The Netherlands

^2^Department of Pathology and Medical Biology, University of Groningen, University Medical Center Groningen, Hanzeplein 1, P.O. Box 30001, 9700 RB Groningen, The Netherlands

^3^Department of Medical Microbiology and Infectious Diseases, Erasmus MC, ‘s Gravendijkwal 230, 3015 CE Rotterdam, The Netherlands

^4^Department of Internal Medicine, Division of Nephrology, University of Groningen, University Medical Center Groningen, Hanzeplein 1, P.O. Box 30001, 9700 RB Groningen, The Netherlands

^5^Department of Rheumatology and Clinical Immunology, University of Groningen, University Medical Center Groningen, Hanzeplein 1, P.O. Box 30001, 9700 RB Groningen, The Netherlands

**Supplementary Figure 1.**

**Supplementary Figure 1.** IgG responses of PR3-ANCA and MPO-ANCA patients and HC to staphylococcal antigens. Bead-based Luminex flow cytometry was used to determine the relative amounts of IgGs against 38 *S. aureus* antigens in sera from 27 PR3-ANCA patients (15 *S. aureus* carriers, 12 non-carriers; one serum/patient), 38 MPO-ANCA patients (27 carriers, 11 non-carriers; 1 serum/patient) and 18 HC (10 carriers with 23 sera, eight non-carriers with 20 sera).
